# A Survey of Tele-Critical Care State and Needs in 2019 and 2020 Conducted among the Members of the Society of Critical Care Medicine

**DOI:** 10.3390/healthcare10081445

**Published:** 2022-08-01

**Authors:** Krzysztof Laudanski, Elizabeth Scruth, Fiona Winterbottom, Mariana Rastrepo, Siddharth Dugar, Vitaly Herasevich, Israel Villanueva, Donna Lee Armaignac, Benjamin K. Scott

**Affiliations:** 1 Department of Anesthesiology and Critical Care, University of Pennsylvania, Philadelphia, PA 19104, USA; 2 Leonard Davis Institute for Health Economics, University of Pennsylvania, Philadelphia, PA 19104, USA; 3 Department of Quality, Data Analytics and Tele-Critical Care, Kaiser Permanente Northern California, Oakland, CA 94612, USA; elizabeth.scruth@kp.org; 4 Critical Care Medicine, Ochsner Health, New Orleans, LA 70121, USA; fwinterbottom@ochsner.org; 5 College of Arts and Sciences, University of Pennsylvania, Philadelphia, PA 19104, USA; rmariana@sas.upenn.edu; 6 Department of Critical Care, Respiratory Institute, Cleveland Clinic, Cleveland, OH 44195, USA; dugars@ccf.org; 7 College of Medicine, Lerner School of Medicine, Case Western Reserve University, Cleveland, OH 44106, USA; 8 Department of Anesthesiology and Perioperative Medicine, Mayo Clinic, Rochester, MN 55902, USA; vitaly@mayo.edu; 9 Intercept Telemed, Inc., Weston, FL 33326, USA; ivillanueva@intercepttelemed.com; 10 Center for Advanced Analytics, Baptist Health South Florida, Miami, FL 33176, USA; donnaar@baptisthealth.net; 11 Department of Anesthesiology and Critical Care, University of Colorado, Denver, CO 80045, USA; benjamin.scott@ucdenver.edu

**Keywords:** Tele-Critical Care, standard of care, competencies, healthcare, innovation, barriers, COVID-19

## Abstract

The study’s objective was to assess facilitators and barriers of Tele-Critical Care (TCC) perceived by SCCM members. By utilizing a survey distributed to SCCM members, a cross-sectional study was developed to analyze survey results from December 2019 and July 2020. SCCM members responded to the survey (*n* = 15,502) with a 1.9% response rate for the first distribution and a 2.54% response rate for the second survey (*n* = 9985). Participants (*n* = 286 and *n* = 254) were almost equally distributed between non-users, providers, users, and potential users of TCC services. The care delivery models for TCC were similar across most participants. Some consumers of TCC services preferred algorithmic coverage and scheduled rounds, while reactive and on-demand models were less utilized. The surveys revealed that outcome-driven measures were the principal form of TCC performance evaluation. A 1:100 (provider: patients) ratio was reported to be optimal. Factors related to costs, perceived lack of need for services, and workflow challenges were described by those who terminated TCC services. Barriers to implementation revolved around lack of reimbursement and adequate training. Interpersonal communication was identified as an essential TCC provider skill. The second survey introduced after the onset pandemic demonstrated more frequent use of advanced practice providers and focus on performance measures. Priorities for effective TCC deployment include communication, knowledge, optimal operationalization, and outcomes measurement at the organizational level. The potential effect of COVID-19 during the early stages of the pandemic on survey responses was limited and focused on the need to demonstrate TCC value.

## 1. Introduction

Tele-Critical Care (TCC) can be described as services that link remote services to a recipient at a local site where physical care is provided using audiovisual communication systems [1]. TCC has been gradually recognized as a valuable means of delivering healthcare services [1,2,3,4,5]. TCC’s growth has been mostly organic, heterogeneous, and often determined by a few key leaders in the field [5,6,7,8]. Lack of reimbursement, medicolegal barriers, cumbersome credentialing processes, and lack of buy-in into technology were identified as significant barriers to the progression of TCC in prior surveys [1,5,9]. Pandemics, technological advances (including software enhancements to TCC platforms), and healthcare policy have also impacted TCC in several ways [10,11,12,13,14]. It remains unclear how much these barriers have changed since the last survey was conducted in 2015, and no studies were re-deployed repetitively to assess the evolution of TCC needs [9,15]. Finally, most surveys are not inclusive of future or past users of TCC, therefore failing to reflect the heterogeneity of critical care healthcare providers in various stages of interaction with TCC technology. An investigation of these two populations may yield valuable insight into the facilitators and barriers to TCC services. Conducting more wide-ranging studies among these cohorts offers a unique insight into barriers to wider implementation and/or the failure of TCC expansion.

In order to address the future needs of TCC, a professional organization formed a TCC Committee to support the growth of TCC [4,8,9,16,17,18]. This survey is designed to attain data regarding TCC’s current landscape, identify practitioners’ needs specific to their roles in healthcare delivery, and guide strategies to support the optimal development of TCC over time. The authors aimed to address the gap of knowledge in the needs of current and future users by developing systematic studies to assess operations, healthcare models, and value-added systems across several TCC domains and providers [1,5,19,20,21,22]. Continuation of these efforts will result in a study assessing needs and barriers in TCC. In this study, the authors investigated unsuccessful programs [5,9,23]. Additionally, the timing of this study allowed for the assessment of the early effects of COVID-19 on TCC, even though it was not the primary goal of the survey [1,2,3]. The survey data will inform TCC stakeholders about the development and design of TCC programs.

## 2. Materials and Methods

The study had an exempt status by the IRB at the University of Pennsylvania.

### 2.1. Survey Development

Several members of a TCC professional committee developed a survey based on their leadership, diversity, and contribution to the field. Members represented academia, consumers, and non-users of TCC. Professional backgrounds included physicians, advanced practice providers, nurses, and pharmacists, thus providing diverse backgrounds for survey input. The survey was developed through four peer-reviewed stages. Members of a subcommittee prepared the first draft based on prior published research and statements [6,9,17,18,20,24,25,26,27]. A second review panel representing the entire committee refined the pre-submission survey format, then two subsequent rounds of modifications occurred prior to survey finalization. During the creation of the survey, several critical determinations were made. First, no definitions were provided for any of the models of TCC delivery in the survey considering the lack of community-wide consensus regarding the nomenclature [4,9]. Providers are defined as the entities delivering TCC to remote locations. Users are those utilizing TCC at the bedside. Non-users are individuals not currently engaged in any form of TCC activity. Second, the number of questions was limited to 25. The authors made the survey an adaptable tool that adjusted questions based on participant responses. Consequently, only essential data were collected as the survey modified itself depending on the answer to a given question (Supplementary Material S1). This allowed the creation of specific sub-surveys for providers considering, implementing, or terminating services.

The final survey was designed to assess the dynamic changes in knowledge, implementation, and needs about TCC. The survey is intended to be deployed every 3–4 years via REDCap [28]. Here, we presented the data from the first edition of the survey.

### 2.2. Distribution of the Survey

The survey was distributed to a professional organization membership in two releases, November/December 2019 and June/July 2020. The second release was approved to investigate the early effects of COVID-19 on TCC, and it is not part of the original design but represents an ad hoc research opportunity. For each release, the survey was sent three times, each time a week apart. The survey was delivered via email to all members requiring membership login to answer. Opening of the email removed that individual from subsequent solicitation, ensuring that the individual could only participate once. The survey was closed a week after the last submission.

**Figure A1:**
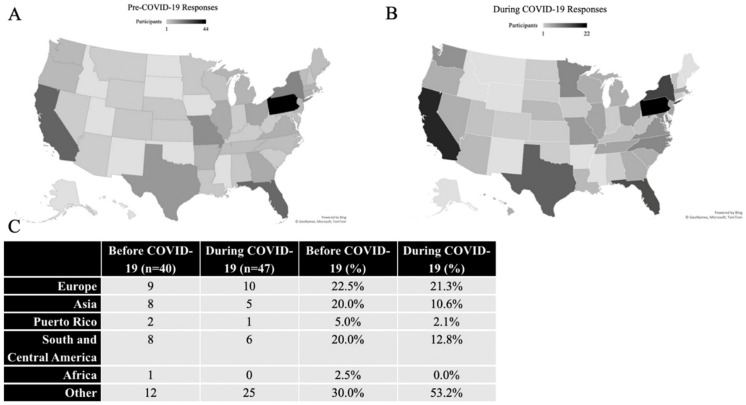
Response distribution at the first (**A**) and the second survey (**B**) with continental breakdown (**C**).

The survey received 286 responses during the first release and 254 during the second release seven months later. The response rate was 1.9% for the first release sent to 15,502 members and 2.54% for the second release after sending the survey to non-responders of the first survey (9985 members). The characteristic of the responders demonstrated a broad representation of healthcare professionals (Appendix A, Table A1). There were minor changes in the territorial distribution of TCC between the two releases, and some of the answers were outside the USA (Appendix A, Figure A1).

### 2.3. Statistical Methods

Descriptive analyses of variables were conducted using frequencies, mean or median depending on the nature of the variables with adequate measures of data distribution. χ2 determined the differences in frequencies. When appropriate, the variables were compared using Student’s unpaired t-test or Mann–Whitney-U statistics. A double-sided *p*-value of less than 0.05 was considered statistically significant for all tests. Statistical analyses were performed using MS Excel (Microsoft, Seattle, WA, USA) and Statistical 11.0 (Stat Soft Inc., Tulsa, OK, USA).

## 3. Results

### 3.1. Overview of the Current State of TCC Services as Reported by Respondents

**Figure A2:**
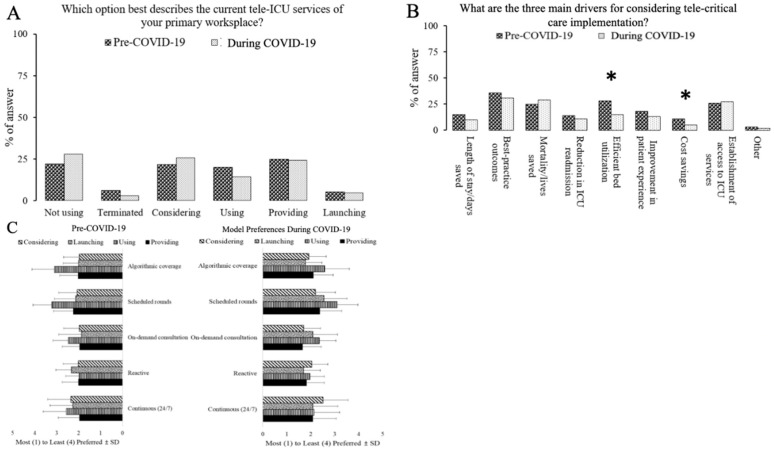
Survey respondents were primarily non-users of users of, providing, or considering TCC services (**A**). The main drivers for considering TCC services (**B**). Preferred TCC care models (**C**). * denotes a statistically significant difference at *p* less than 0.05.

Respondents represented all types of institutions (Veterans Affairs, academic, community, not-for-profit, and for-profit) across urban and rural settings. Table 1 describes the respondents’ healthcare roles and specialties, broken down by respondent category and the total sample. In summary, the “not using and not considering TCC” (24.6%), “considering TCC” (23.3%), “providing TCC” (24.4%), and “utilizing TCC” (17.2%) were approximately evenly distributed categories, with “currently launching” (5.0%) and “had TCC in the past” (4.6%) comprising the minority of respondents. Across all categories, most respondents were physicians (70.9%), followed by registered nurses (11.8%) and nurse practitioners (5.6%). The most popular specialty was Internal Medicine/Pulmonology. There were no significant differences between groups of participants that were users, providers, or non-users of TCC services, with a minority of responders launching or terminating services in December 2019 or in June 2020 (Appendix A, Figure A2A). Increases in non-users and those considering launching services, and decreases in those considering terminating services, were observed trends between the two survey releases, but the differences were non-significant. The top three drivers for establishing TCC services were: the implementation of best practices, mortality/lives saved, and establishing access to ICU services (Appendix A, Figure A2B). Cost savings and efficient bed utilization were less stressed in the second survey, although this was not statistically significant.

TCC care delivery models use was similar across all three stages of service delivery (considering services, launching services, and active users), with the marked exception of certain users of TCC services putting more importance on algorithmic coverage (early warning system alerts) and scheduled rounds (Appendix A, Figure A2C). In contrast, reactive (at time of emergency or patient deterioration) and on-demand models were less utilized (Appendix A, Figure A2C). In addition, TCC care delivery model preferences stayed consistent across both survey releases (data not shown).

### 3.2. Operational Aspects of Providers and Users of TCC Services

Both users and providers reported above-average satisfaction for TCC services (Users = 60.2 + 19.54 vs. Providers = 65 + 20.89; *p* = ns) with no effect of time between surveys. TCC services were utilized on average for 5.4 + 3.04 ($\bar{x}$ + SD) years for current users. Providers offering TCC services reported being on the market for 5.4 + 5.61 ($\bar{x}$ + SD) years.

**Figure 1:**
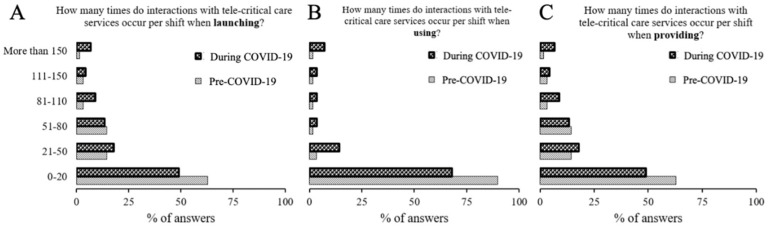
Description of engagement frequency across providers and users across different stages of Tele-CCM services. There was a difference in length of the interactions between individuals launching services (**A**) and providing services (**C**) as compared to users. However, users reported longer encounters in the edition released in June 2020 (**B**).

Most centers contracting TCC services utilized them less than 20 times per night (Figure 1A,B). Providers reported significantly more interactions with some increase in interaction length in the second edition of the survey (Figure 1A–C). Most users (63%) and providers (58%) stated 1:50 as the optimal ratio between providers and patients, with users (40.7%) shifting towards a 1:100 provider:patient ratio after deploying the survey the second time (data not shown).

**Figure A3:**
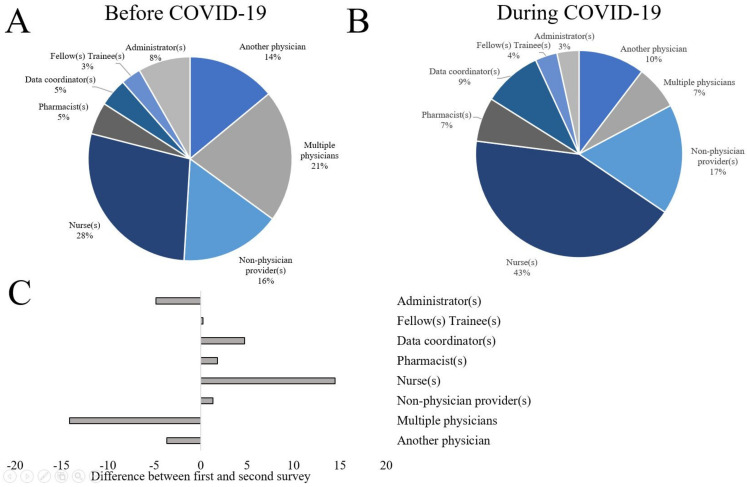
Change in survey respondent composition between the first (**A**) and second (**B**) survey deployment with relative changes between them (**C**).

The usual composition of a TCC team consists of MDs and RNs, while advanced practice providers, pharmacists, respiratory therapists, and data coordinators were in the minority. However, participation of non-physician providers significantly increased after the second survey (Appendix A, Figure A3A–C).

### 3.3. Evaluation of TCC Performance by Survey Respondents

**Figure 2:**
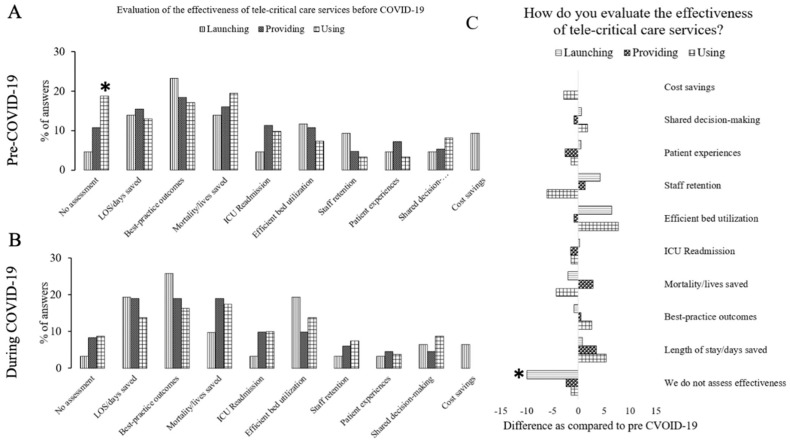
Evaluation of the value delivered by the Tele-CCM. Reported quality of metric of Tele-Critical Care delivery in December 2019 (**A**) and in June 2020 (**B**) with a relative difference (**C**) were significant for an increase in reported assessment in users after the emergence of COVID (* *p* < 0.05).

The survey of TCC services’ effectiveness showed that outcome-driven measures were most important, including implementing best practices, length of stay, lives saved, and mortality (Figure 2A). An interesting observation was that 20% of respondents using the TCC services were unaware of the processes used to evaluate their effectiveness while providing the services before COVID-19 (Figure 2C). For example, cost savings were evaluated during TCC initiation and were not frequently reassessed. During the COVID-19 pandemic, significantly fewer participants reported the need to assess TCC effectiveness and value compared to pre-COVID-19 (*p* = 0.03; Figure 2B,C).

### 3.4. Perception of Barriers and Facilitators in TCC Development

Barriers to the widespread utilization of TCC included a lack of reimbursement and adequate training (Table 1). Patient privacy was of limited concern during both survey periods, whereas degradation of autonomy was more frequently highlighted by TCC providers when compared to individuals with no prior TCC knowledge (Table 1).

In addition, non-users of TCC services requested more educational opportunities, while users stressed novel research. (Table 2). These facilitators were coupled with the perception of communication skills as essential for TCC providers, followed by broad ICU and healthcare operations (Table 2).

### 3.5. Responses by Individuals Contemplating or Introducing TCC Services

In December 2019, approximately 30% of survey respondents were considering (*n* = 61) TCC services, with the majority being in Asia (6.6%), CA (9.8%), MO (6.6%), PA (6.6%), and South/Central America (6.6%). In June 2020, a similar percentage of respondents reported considering (*n* = 65) services, with most of the respondents reported being from the following regions: other (21.5%), CA (13.8%), NY (9.2%), and TX (9.2%). For both time points, most respondents reported being engaged in the “discussion regarding the implementation of the services” stage (PreCOVID-19 = 41.4% + 18.7 vs. During COVID-19 = 36.4% + 16.3; *p* = ns).

**Figure A4:**
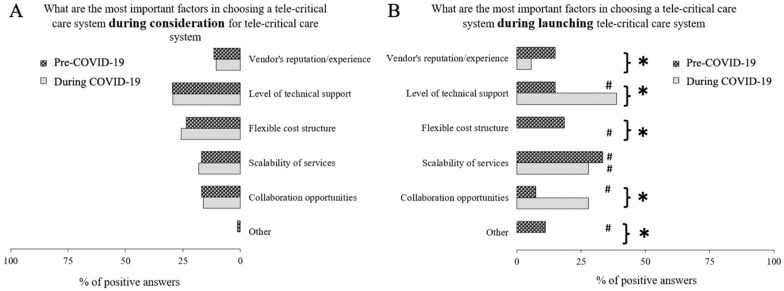
While COVID-19 had little impact on critical factors for Tele-Critical Care (**A**), there was a significant change in priorities on the practices launching the TCC before and after COVID-19 (**B**). * denotes the statistically significant difference between and after the emergence of COVID-19; # denotes the difference between considering and launching the services in respective domains in the 2019 or 2020 survey.

In December 2019, respondents reporting TCC implementation (*n* = 15) were in TX (26.7%), CA (26.7%), and VT (13.3%). In June 2020, respondents reporting TCC implementation (*n* = 12) were in PA (16%), CA (8%), TX (8%), and MN (8%). The expected difficulty TCC implementation was perceived as almost identical before and during COVID-19 (PrePerception = 31.1 + 18.7 vs. During Perception = 31.8 + 13.46 [%]). In December 2019, the top four reasons for initiation of TCC services were “implementation of best practices”, “efficient bed management”, “access to TCC services”, and “improved survival”. In June 2020, the drivers for initiation of TCC services fluctuated non-significantly with the following responses: “lives saved” and “establishing the access to the ICU services” (data not shown). For the first survey, most of the respondents used locally developed platforms (26.7%), followed by Philips eCare Manager™ (20%), EPIC™ (13.3%), and others (40%). The second survey demonstrated a statistically non-significant increase in percentages of locally developed platforms (44.4%), followed by Philips eCare Manager (33.3%). The most important drivers for choosing the platform in the first survey were technological stability and interoperability (33.3%) (Appendix A, Figure A4A). In June 2020, the most important driver for choosing the platform in the second survey was the level of technical support (28%) (Appendix A, Figure A4B).

### 3.6. Insight into Termination of TCC Services

Before the pandemic, 6% of the respondents (*n* = 26) terminated TCC services. Most of the terminations occurred in ME (29.4%) and CA (11.8%), with 30.8% being terminated due to costs, followed by 23.1% being terminated due to “system hospital change”. The average length of a TCC program was 5.4 years before termination. In June 2020, approximately 3% (*n* = 8) of responses noted the termination of TCC services. Twenty-five percent of terminations occurred in Europe, with the remainder being equally distributed across IL, TN, FL, South and Central America, TX, and WA, with 36.4% of respondents indicating that cost was the primary factor for termination, with no appreciable need for services (27.3%) or change in the workflow (18.2%).

## 4. Discussion

Previous papers described facilitators and barriers of TCC models [2,5,8]. The TCC service models were relatively homogenous between providers and consumers, which is consistent with prior surveys [2,5,8]. This may reflect operational flexibility, the search for an optimal model, or a lack of an exact strategy for TCC implementation within a particular system [7,17,22,29,30]. The implementation drivers seem to align well with the existing literature demonstrating best practices, length of stay, bed utilization, and improvements in mortality [27,30,31,32,33,34]. Interestingly, a significant percentage of providers were not aware of the performance measures of their own TCC service in service in 2019; this improved somewhat in 2020. This lack of clear performance measures is worrisome as it may indicate communication barriers leading to TCC optimization or termination [6,35,36,37].

The was a consensus for a 1:50 to 1:150 ratio between providers and patients with significant changes in the second survey, suggesting improvement in utilization of TCC services [2,7,22,31]. These ratios are significantly higher than those suggested in the Pronovost et al. study from 2003 and by TCC platform manufacturers [9,22,29,32]. The survey demonstrated a diverse TCC staff composition, including physicians, advanced practice providers, nurses, pharmacists, support staff, and information services [2,5,9,19]. Future studies are necessary to assess staffing models of TCC in the context of workflow implementation [31,38,39]. In alignment with the pre-existing literature, effective communication was highlighted as an important skill in the survey, with insufficient communication mentioned as the primary reason for the lack of success or termination of TCC care services [17,20,31,39,40,41].

Opportunities exist to develop standardized performance measures and implementation strategies for TCC. Respondents in this survey rated deployment of TCC as extremely challenging, requiring months to implement, lack of goals and measurable outcomes for success, and an overall knowledge gap [5,17,31,42,43]. The dominant challenge noted in our study complicating the implementation of TCC is the deficiency of fundamental knowledge of the function and role of TCC among non-TCC users. Organizational barriers noted in earlier surveys, such as cost, were also noted in this survey, which is somewhat disappointing, signaling that little progress has been made in understanding and resolving these barriers over the past decade [8,9,16,24,25,26,39]. A narrative review discussed the implementation and subsequent operating costs of TCC, with few studies reporting actual financial savings or a thorough description of accounting methods [39].

Termination of the TCC services occurred primarily due to operational reasons. Cost, poorly designed workflows, and lack of discernable impact on care were cited as the reasons and may result from an imperfect implementation process [24,44].

The surveys were disseminated at the beginning of the COVID-19 pandemic, which was not the original research design but did provide an opportunity to assess the effect of the COVID-19 pandemic on TCC [9,16]. TCC was suggested as an optimal solution for maximizing the demand–supply imbalance during a pandemic [5,8,23,33]. A limited effect was observed, which focused mostly on defining the value of TCC during COVID-19 and increased participation of advanced practice providers. The second survey was deployed very early after the initial COVID-19 surge, with potential response bias from mature TCC centers cautioning against definite conclusions [45,46].

Limitations of this research include the survey method of data collection and a low response rate (~2%). In-depth analyses of different subgroups were not conducted; however, study findings align with prior results [9,16]. The investigators did not define several items in the survey as definitions relating to TCC vary but allowed for organic participant interpretation of survey questions [9,16,39,47]. The survey was distributed exclusively among the members of a large medical professional organization in the USA, and it represents a particular group of professionals, which could bias results. In the next iteration of the survey, additional organizations should be included. The survey results should not be used to estimate the effect of COVID-19 on TCC, as it was deployed early in the pandemic [23,45,46].

## 5. Conclusions

In summary, findings from this study suggest a need to develop a standardized approach to TCC implementation, including training, communication, and outcome-driven performance measures. This approach would allow for the development of common quality measures for benchmarking across all TCCs. Robust training programs are needed to resolve issues related to perceived lack of need for services and workflow challenges.

## Figures and Tables

**Table 1:** Assessment of the significant barriers to the development of Tele-Critical Care services demonstrated concerns for privacy and degradation of autonomy. Bolded numbers signify statistical differences between groups. * denotes statistically significant differences before and during COVID-19. # denotes statistically significant differences between individuals with or without Tele-Critical Care experience.

Concerns Regarding Tele-Critical Care Use		% December 2019	% June 2020
No TCC Experience: *n* = 353 before COVID-19, *n* = 340 December 2019 TCC Experience: *n* = 449 before COVID-19, *n* = 289 June 2020			
**Costs**	No TCC experience	14.4%	16.8%
	TCC experience	13.4%	15.2%
**Privacy**	No TCC experience	**5.9%**	**1.6% #**
	TCC experience	4.2%	3.5%
**Lack of reimbursement**	No TCC experience	18.7%	15.0%
	TCC experience	19.8%	22.5%
**Legal responsibility**	No TCC experience	21.5%	**23.5%**
	TCC experience	18.9%	15.2% *
**Lack of TCC knowledge**	No TCC experience	13.3%	10.6%
	TCC experience	12.5%	12.8%
**Degradation of autonomy**	No TCC experience	**9.6%**	**8.5%**
	TCC experience	16.9% *	15.9% *
**Lack of TCC training**	No TCC experience	16.4%	17.9%
	TCC experience	14.2%	14.9%

**Table 2:** Assessment of critical skills for effective delivery of Tele-Critical Care services revealed interpersonal skills as the critical skills to be an effective Tele-CCM provider. Bolded numbers signify statistical differences between groups. * denotes statistically significant differences between individuals with or without Tele-Critical Care experience. # denotes statistically significant difference before and during COVID-19.

Requirements Necessary to be Effective Tele-Intensivists		% December 2019	% June 2020
No TCC Experience: *n* = 241 before COVID-19, *n* = 264 December 2019 TCC experience: *n* = 325 before COVID-19, *n* = 232 June 2020			
**Broad ICU knowledge**	No TCC experience	15.8%	15.2%
	TCC experience	20.0%	16.4%
**TCC experience**	No TCC experience	15.4%	13.3%
	TCC experience	12.3%	11.6%
**Communication skills**	No TCC experience	30.7%	32.6%
	TCC experience	32.0%	31.0%
**Interpersonal skills**	No TCC experience	11.2%	**10.6%**
	TCC experience	**11.4%**	17.7% # *
**Knowledge of healthcare systems operations**	No TCC experience	12.4%	13.6%
	TCC experience	14.5%	9.5%
**Technological skills**	No TCC experience	8.3%	11.0%
	TCC experience	7.1%	12.1%
**I do not know**	No TCC experience	6.2%	3.8%
	TCC experience	2.8%	1.7%

## Data Availability

The datasets used and/or analyzed during the current study are available from the corresponding authors on reasonable request after approval from the SCCM.

## Supplementary Materials

The following supporting information can be downloaded at: https://www.mdpi.com/article/10.3390/healthcare10081445/s1, Supplementary Material S1: Data Dictionary Codebook’.

## Appendix A

**Table A1:** Characteristics of the study sample.

Primary Clinical Affiliation	*n* = 454 December 2019 % (*n*)	*n* = 411 June 2020 % (*n*)
Veteran’s Administration	1.1% (5)	1.7% (7)
Academic	38.3% (174)	36.5% (150)
Community	16.1% (73)	17.8% (73)
Nonprofit	17.0% (77)	16.3% (67)
For-profit	3.5% (16)	3.6% (15)
Urban	11.0% (50)	9.9% (41)
Rural	3.1% (14)	2.2% (9)
Within-state	5.5% (25)	6.8% (28)
Cross-state	3.3% (15)	3.4% (14)
Cross-country	1.1% (5)	1.7% (7)
Healthcare role	*n* = 286	*n* = 254
Physician	194 (67.8%)	186 (73.2%)
Nurse practitioner	16 (5.6%)	14 (5.5%)
Physician Assistant	8 (2.8%)	9 (3.5%)
Registered nurse	38 (13.3%)	25 (9.8%)
Respiratory Therapist	4 (1.4%)	0 (0%)
Pharmacist	16 (5.6%)	10 (3.9%)
Emergency medical services	1 (0.35%)	1 (0.39%)
Research	5 (1.7%)	1 (0.39%)
Other	4 (1.4%)	8 (3.1%)
Physician Specialty	*n* = 194	*n* = 254
Internal medicine/pulmonology	66 (34.0%)	46 (18.1%)
Anesthesiology	29 (14.9%)	11 (4.3%)
Cardiology	10 (5.2%)	3 (1.2%)
Neurology	6 (3.1%)	5 (1.9%)
Emergency physician	32 (16.5%)	3 (1.2%)
Surgery	28 (14.4%)	24 (9.4%)
Pediatrics	1 (0.52%)	41 (16.1%)
Hospitalist	22 (11.3%)	2 (0.79%)
Other	92 (47.4%)	119 (46.9%)

## References

[1] Subramanian S., Pamplin J.C., Hravnak M., Hielsberg C., Riker R., Rincon F., Laudanski K., Adzhigirey L.A., Moughrabieh M.A., Winterbottom F.A. (2020). Tele-Critical Care: An Update From the Society of Critical Care Medicine Tele-ICU Committee. Crit. Care Med..

[2] Arneson S.L., Tucker S.J., Mercier M., Singh J. (2020). Answering the Call: Impact of Tele-ICU Nurses during the COVID-19 Pandemic. Crit. Care Nurse.

[3] Al-Saadi M.A., Wright J.U., Masud F.N. (2020). Tele-ICU: A key to residents’ role in the intensive care unit during COVID-19 pandemic. Clin. Teach..

[4] Williams L.-M.S., Johnson E., Armaignac D.L., Nemeth L.S., Magwood G.S. (2019). A Mixed Methods Study of Tele-ICU Nursing Interventions to Prevent Failure to Rescue of Patients in Critical Care. Telemed. J. e-Health.

[5] Lilly C.M., Greenberg B. (2020). The Evolution of Tele-ICU to Tele-Critical Care. Crit. Care Med..

[6] Fusaro M.V., Becker C., Scurlock C. (2019). Evaluating Tele-ICU Implementation Based on Observed and Predicted ICU Mortality: A Systematic Review and Meta-Analysis. Crit. Care Med..

[7] Khunlertkit A., Carayon P. (2013). Contributions of tele-intensive care unit (Tele-ICU) technology to quality of care and patient safety. J. Crit. Care.

[8] Srinivasan S.R. (2020). Editorial: Tele-ICU in the Age of COVID-19: Built for This Challenge. J. Nutr. Health Aging.

[9] Lilly C.M., Thomas E.J. (2010). Tele-ICU: Experience to date. J. Intensive Care Med..

[10] Cawley J.F. (2021). The Ten Year War: Obamacare and the Unfinished Crusade for Universal Coverage. J. Physician Assist. Educ..

[11] Thind A., Hagigi F. (2010). Obamacare: Next steps in US healthcare reform. Natl. Med. J. India.

[12] Orso F., Migliorini M., Herbst A., Ghiara C., Virciglio S., Camartini V., Tognelli S., Lucarelli G., Fortini G., Pratesi A. (2020). Protocol for Telehealth Evaluation and Follow-up of Patients with Chronic Heart Failure during the COVID-19 Pandemic. J. Am. Med. Dir. Assoc..

[13] Huckman R. (2020). What Will U.S. Health Care Look Like after the Pandemic. Harvward Bus. Rev..

[14] Herasevich V., Subramanian S. (2019). Tele-ICU Technologies. Crit. Care Clin..

[15] Ward M.M., Ullrich F., Potter A.J., MacKinney A.C., Kappel S., Mueller K.J. (2015). Factors Affecting Staff Perceptions of Tele-ICU Service in Rural Hospitals. Telemed. J. e-Health.

[16] Lilly C.M., Fisher K.A., Ries M., Pastores S., Vender J., Pitts J.A., Hanson W.C. (2012). A national tele-ICU survey. Chest.

[17] Becker C.D., Fusaro M.V., Scurlock C. (2019). Deciphering factors that influence the value of tele-ICU programs. Intensive Care Med..

[18] Zapka J., Simpson K., Hiott L., Langston L., Fakhry S., Ford D. (2013). A mixed methods descriptive investigation of readiness to change in rural hospitals participating in a tele-critical care intervention. BMC Health Serv. Res..

[19] Griffiths C.L., Kosmisky D.E., Everhart S.S. (2020). Characterization of dayshift tele-ICU pharmacist activities. J. Telemed. Telecare.

[20] Venditti A., Edelstein T., Brock A.J. (2015). Transformation of ICU and tele-ICU annual competencies. Nurs. Manag..

[21] Brindise T., Baker M.P., Juarez P. (2015). Development of a Tele-ICU Postorientation Support Program for Bedside Nurses. Crit. Care Nurse.

[22] Morse H.G., Hunt B., Wheeler C., Kopec I., Hand S., Somers W., Tillirson M. (2014). Tele-Intensivist Augmented Critical Care: Report of Three-Year Experience with a Remote Tele-ICU System. J. South Carol. Med. Assoc..

[23] Ramakrishnan N., Tirupakuzhi Vijayaraghavan B.K., Venkataraman R. (2020). Breaking Barriers to Reach Farther: A Call for Urgent Action on Tele-ICU Services. Indian J. Crit. Care Med..

[24] Young L.B., Chan P.S., Cram P. (2011). Staff acceptance of tele-ICU coverage: A systematic review. Chest.

[25] Rufo R. (2011). Using the Tele-ICU care delivery model to build organizational performance, part 1. Crit. Care Nurs. Q..

[26] Ries M. (2016). Evaluating Tele-ICU Cost--An Imperfect Science. Crit. Care Med..

[27] Becker C.D., Fusaro M.V., Al Aseri Z., Millerman K., Scurlock C. (2020). Effects of Telemedicine ICU Intervention on Care Standardization and Patient Outcomes: An Observational Study. Crit. Care Explor..

[28] Harris P.A., Taylor R., Minor B.L., Elliott V., Fernandez M., O’Neal L., McLeod L., Delacqua G., Delacqua F., Kirby J. (2019). The REDCap consortium: Building an international community of software platform partners. J. Biomed. Inform..

[29] Ramnath V.R., Khazeni N. (2014). Centralized monitoring and virtual consultant models of tele-ICU care: A side-by-side review. Telemed. J. e-Health.

[30] Van Gent J.-M., Davis K.L., Henry N., Zander A.L., Kuettel M.A., Edson T., Nelson T.J., Tadlock M.D. (2018). The Initial Impact of Tele-Critical Care on the Surgical Services of a Community Military Hospital. Mil. Med..

[31] Becker C.D., Yang M., Fusaro M., Fry M., Scurlock C.S. (2019). Optimizing Tele-ICU Operational Efficiency Through Workflow Process Modeling and Restructuring. Crit. Care Explor..

[32] Pronovost P.J., Angus D.C., Dorman T., Robinson K.A., Dremsizov T.T., Young Y.L. (2002). Physician staffing patterns and clinical outcomes in critically ill patients: A systematic review. JAMA.

[33] Wood D. (2011). Tele-ICU saves money as well as lives. Telemed. J. e-Health.

[34] Lilly C.M., Zubrow M.T., Kempner K.M., Reynolds H.N., Subramanian S., Eriksson E.A., Jenkins C.L., Rincon T.A., Kohl B.A., Groves R.H. (2014). Critical care telemedicine: Evolution and state of the art. Crit. Care Med..

[35] Peltan I.D. (2019). ICU Telemedicine and Mortality: If It Ain’t Broke, Fixing It Won’t Help. Crit. Care Med..

[36] Bose S., Singh V.K. (2008). Barriers to effective communication in developing world intensive care units. Crit. Care Med..

[37] Trevick S., Kim M., Naidech A. (2016). Communication, Leadership, and Decision-Making in the Neuro-ICU. Curr. Neurol. Neurosci. Rep..

[38] Essay P., Shahin T.B., Balkan B., Subbian V. (2019). The Connected Intensive Care Unit Patient: Exploratory Analyses and Cohort Discovery from a Critical Care Telemedicine Database. JMIR Med. Inform..

[39] Vranas K.C., Slatore C.G., Kerlin M.P. (2018). Telemedicine Coverage of Intensive Care Units: A Narrative Review. Ann. Am. Thorac. Soc..

[40] Hoonakker P.L., Pecanac K., Brown R.L., Carayon P. (2017). Virtual collaboration, satisfaction, and trust between nurses in the tele-ICU and ICUs: Results of a multilevel analysis. J. Crit. Care.

[41] Chu-Weininger M.Y.L., Wueste L., Lucke J.F., Weavind L., Mazabob J., Thomas E.J. (2010). The impact of a tele-ICU on provider attitudes about teamwork and safety climate. Qual. Saf. Health Care.

[42] Lilly C.M., Cody S., Zhao H., Landry K., Baker S.P., McIlwaine J., Chandler M.W., Irwin R.S., University of Massachusetts Memorial Critical Care Operations Group (2011). Hospital mortality, length of stay, and preventable complications among critically ill patients before and after tele-ICU reengineering of critical care processes. JAMA.

[43] Hassan E. (2018). Tele-ICU and Patient Safety Considerations. Crit. Care Nurs. Q..

[44] Moeckli J., Cram P., Cunningham C., Reisinger H.S. (2013). Staff acceptance of a telemedicine intensive care unit program: A qualitative study. J. Crit. Care.

[45] Heinonen K., Strandvik T. (2021). Reframing service innovation: COVID-19 as a catalyst for imposed service innovation. J. Serv. Manag..

[46] Ramadi K.B., Nguyen F.T. (2021). Rapid crowdsourced innovation for COVID-19 response and economic growth. NPJ Digit. Med..

[47] Caples S.M. (2019). Intensive Care Unit Telemedicine Care Models. Crit. Care Clin..

