# Supplementary material for: A Survey of Tele-Critical Care State and Needs in 2019 and 2020 Conducted among the Members of the Society of Critical Care Medicine
Source: Healthcare (Basel). 2022 Aug 1;10(8):1445. doi: 10.3390/healthcare10081445 (PMC9408319; doi:10.3390/healthcare10081445)
Supplement: Supplementary file 1 [file healthcare-10-01445-s001.zip › healthcare-1809856-supplementary.pdf]

Tele-Critical Care Study 2

Data Dictionary Codebook

25/08/2020 10:12 AM

#	Variable / Field name	Field label<br>*Field Note*	Field Attributes (Field type, Validation, Choices, Calculation, etc.)
Instrument: **teleicu** Version: **(original)**			Enabled as Survey
1	consent_1	Section Header: *Tele-Critical Care Survey Respondent Consent*<br>Please review the informed consent document.	Descriptive Text
2	consentyn	Have you read the consent and agree to participate in the study?	Radio Buttons, Required<br>1 Yes<br>2 No
3	Part 1 of a 2-part survey. We appreciate your consent. Part 1 will determine the subsequent final survey.	Section Header: *Background and Tele-Critical Care Status*<br>*(Branching logic expression: [consentyn]= 1)*<br>Part 1 of a 2-part survey. We appreciate your consent. Part 1 will determine the subsequent final survey.	New Section
4	zip	What is the zip code of your work location? (Enter a three-digit code outside the United States: 001 for Europe, 002 for Asia, 003 for South and Central America, 005 for Africa, or 006 for Other).	Text Box
5	icu_affiliation	Which option best describes your primary clinical affiliation? (Select all that apply).	Checkboxes<br>1 Veterans Administration<br>2 Academic<br>3 Community<br>4 Nonprofit<br>5 For-profit<br>6 Urban<br>7 Rural<br>8 Within-state

			9 Cross-state<br>10 Cross-country
6	role_healthcare	What is your role in healthcare?	Drop-down<br>1 Physician<br>2 Nurse practitioner<br>3 Physician assistant<br>4 Registered nurse<br>5 Respiratory therapist<br>6 Pharmacist<br>7 Emergency medical services<br>8 Research<br>9 Other
7	oth_role	Please specify and describe "Other" role.	Notes Box, Branching logic expression: [role_healthcare]='9'
8	role_healthcare_spc	What is your specialty?	Drop-down, Branching logic expression: [role_healthcare] = '1'<br>1 Internal medicine /pulmonology<br>2 Anesthesiology<br>9 Cardiology<br>3 Neurology<br>4 Emergency physician<br>5 Surgery<br>6 Pediatrics<br>7 Hospitalist<br>8 Other
9	oth_spec	Please specify and describe "Other" specialty.	Text Box
10	utlz	Which option best describes the current Tele-Critical Care services of your primary workplace?	Radio Buttons, Required<br>1 Not using and not considering Tele-Critical Care services<br>2 Not using but considering Tele-Critical Care services

			3 Currently launching Tele-Critical Care services<br>4 Had Tele-Critical Care services in the past<br>5 Currently providing Tele-Critical Care services<br>6 Currently using Tele-Critical Care services
11	needs	In your opinion, what are the most significant needs for Tele-Critical Care development? Select up to three.	Checkboxes<br>1 Education on Tele-Critical Care establishment and operations<br>2 Research on Tele-Critical Care matters<br>3 Collaboration and networking with peers in the Tele-Critical Care field<br>4 Credentialing and cross-state licensing in regard to Tele-Critical Care<br>5 Advocacy regarding legislative measures pertaining to Tele-Critical Care<br>6 Investigation of Tele-Critical Care delivery practices<br>7 Development of Tele-Critical Care best practices, quality metrics, and standards of practice
12	difference	In your opinion, which two requirements are necessary to be an effective Tele-Intensivist compared to being an in-person Intensivist?	Checkboxes<br>1 Broader knowledge of critical care<br>2 Depth of Tele-Critical Care experience<br>3 Communications skills<br>4 Interpersonal skills<br>5 In-depth knowledge of healthcare systems operations<br>6 Technological skills

			7 I do not know
13	concern	What are the three major concerns regarding the use of Tele-Critical Care?	Checkboxes<br>1 Costs<br>2 Privacy<br>3 Lack of reimbursement parity by insurance providers<br>4 Legal responsibility<br>5 Lack of knowledge about Tele-Critical Care<br>6 Perceived degradation of autonomy<br>7 Lack of Tele-Critical Care-specific training
14	sccm	How can SCCM's Tele-Critical Care Committee support your Tele-Critical Care needs? Select the three that are most important to you.	Checkboxes<br>1 Develop training in skills pertaining to receiving or delivering Tele-Critical Care services<br>2 Create a data repository of critical care patients including Tele-Critical Care-specific data<br>3 Advocate for legislative measures related to Tele-Critical Care<br>4 Facilitate the development of standards for Tele-Critical Care delivery<br>5 Facilitate the development of standards for Tele-Critical Care research<br>6 SCCM has no role in Tele-Critical Care<br>7 Other
15	suggestion1	Please specify and describe "other" areas where the Tele-Critical Care Committee can support your Tele-Critical Care needs.	Text Box, Branching logic expression: [sccm(7)] = '1'
16	involvement	In which domains is the work of	Checkboxes

		the Tele-Critical Care Committee falling short of your expectations? Select the two most problematic.	1 Lack of relevance to my current practice<br>2 Lack of clear mission<br>3 Lack of awareness of committee activities<br>4 Lack of interest of SCCM community in committee offerings<br>5 Inability to communicate with the committee<br>6 None
17	textother	Please share any additional comments you may wish to communicate to the Tele-Critical Care Committee.	Text Box
18	advo	Section Header: *Not using and not considering using Tele-Critical Care services (Branching logic expression: [utlz]=1)*<br>How strongly would you advocate for Tele-Critical Care services implementation?	Slider, Slider labels: Not at all, Neutral, Very strongly
19	barrier	What is the main barrier to your organization's participation in a Tele-Critical Care program?	Checkboxes<br>1 Upfront capital cost of implementation<br>2 Security of maintenance of ongoing operational funding<br>3 Lack of knowledge and appreciation of benefits attainable through Tele-Critical Care<br>4 Organizational barriers to implementation<br>5 Other
20	frtext	Please specify and describe "other" barriers.	Notes Box, Branching logic expression: [barrier(5)] = '1'
21	implenetcons	Section Header: *Not using but considering Tele-Critical Care services (Branching logic expression: [utlz]=2)*	Slider, Slider labels: Not likely, Likely, For sure

		How likely are you to implement Tele-Critical Care services in the future?	
22	long_v2_v2cons	Where are you in the process of considering the implementation of Tele-Critical Care services?	Slider, Slider labels: Not considering, In discussion w/ leadership re implementation, Decision made
23	differentcons	How difficult do you expect the implementation of Tele-Critical Care to be?	Slider, Slider labels: Very difficult, As other programs in our location, Very easy
24		Please indicate your Tele-Critical Care model preferences	Matrix Of Fields
25	item1_sec4	Continuous (24/7)	Matrix Of Fields: Please indi... Radio Buttons<br>1 Most preferred<br>2 Somewhat preferred<br>3 Neutral<br>4 Least preferred
26	item2_sec4	Reactive (in response to an acute physiologic issue	Matrix Of Fields: Please indi... Radio Buttons<br>1 Most preferred<br>2 Somewhat preferred<br>3 Neutral<br>4 Least preferred
27	item3_sec4	On-demand critical care consultation	Matrix Of Fields: Please indi... Radio Buttons<br>1 Most preferred<br>2 Somewhat preferred<br>3 Neutral<br>4 Least preferred
28	item4_sec4	Scheduled Tele-Critical Care rounds at a particular time	Matrix Of Fields: Please indi... Radio Buttons<br>1 Most preferred<br>2 Somewhat preferred<br>3 Neutral<br>4 Least preferred

29	item5_sec4	Utilization of Clinical Decision Support – Attention and treatment response prn to algorithmic severity/acuity scores, smart alerts, deterioration scores	Matrix Of Fields: Please indi... Radio Buttons<br>1 Most preferred<br>2 Somewhat preferred<br>3 Neutral<br>4 Least preferred
30	choosesystemcons	What are the three most important factors in choosing a Tele-Critical Care system?	Checkboxes<br>1 Vendor's reputation and level of experience<br>2 Level of technical support<br>3 Costs<br>4 Scalability of Tele-Critical Care services<br>5 Broadly understood collaboration opportunities with Tele-Critical Care services<br>6 Other
31	otherfactorcons	Please specify and describe the "other" factors?	Notes Box, Branching logic expression: [choosesystemcons (6)] = '1'
32	main_drivers_imp	What are the three main drivers for considering Tele-Critical Care implementation?	Checkboxes<br>1 Length of stay/days saved<br>2 Best-practice outcomes<br>3 Mortality/lives saved<br>4 Reduction in ICU readmission<br>5 Efficient bed utilization<br>6 Improvement in patient experience<br>7 Cost savings<br>8 Establishment of access to ICU services<br>9 Other
33	other	Please specify and describe "other" drivers in Tele-Critical	Notes Box, Branching logic expression: [main_drivers_imp(9)]

		Care implementation.	= '1'
34	long_v2_v2	Section Header: *Currently launching a Tele-Critical Care system (Branching logic expression: [utlz]=3)*<br>For how many months have you been considering developing and implementing a Tele-Critical Care system?	Number Box (Decimal), Min: 0, Max: 50
35	implenet	For how many months have you been developing and implementing a Tele-Critical Care system?	Number Box (Decimal), Min: 0, Max: 100
36	different	How different was the actual process of implementing a Tele-Critical Care system from your initial expectations?	Slider, Slider labels: Much harder than expected, as expected, much easier than expected
37		Please indicate your Tele-Critical Care model preferences	Matrix Of Fields
38	item1_sec5	Continuous (24/7)	Matrix Of Fields: Please indi... Radio Buttons<br>1 Most preferred<br>2 Somewhat preferred<br>3 Neutral<br>4 Least preferred
39	item2_sec5	Reactive (in response to an acute physiologic issue)	Matrix Of Fields: Please indi... Radio Buttons<br>1 Most preferred<br>2 Somewhat preferred<br>3 Neutral<br>4 Least preferred
40	item3_sec5	On-demand Tele-Critical Care consultation	Matrix Of Fields: Please indi... Radio Buttons<br>1 Most preferred<br>2 Somewhat preferred<br>3 Neutral<br>4 Least preferred

41	item4_sec5	Scheduled Tele-Critical Care rounds at a particular time	Matrix Of Fields: Please indi...<br>Radio Buttons<br>1 Most preferred<br>2 Somewhat preferred<br>3 Neutral<br>4 Least preferred
42	item5_sec5	Utilization of Clinical Decision Support- Attention and treatment response prn to algorithmic severity/acuity scores, smart alerts, deterioration scores	Matrix Of Fields: Please indi...<br>Radio Buttons<br>1 Most preferred<br>2 Somewhat preferred<br>3 Neutral<br>4 Least preferred
43	relationship_v2_v2	How would you describe the future working relationship between Tele-Critical Care and bedside ICU clinicians? 1. Tele-Critical Care standards and goals of care developed and evaluated by Tele-Critical Care only. 2. Tele-Critical Care standards and goals of care developed and evaluated by bedside ICU clinicians only. 3. Tele-Critical Care standards and goals of care developed and evaluated by Tele-Critical Care and bedside ICU clinicians collaboratively.	Slider, Slider labels: ICU driven, ICU and tele-critical care driven equally, Tele-critical care driven
44	interaction_v2_v2	Which platform did you decide to use?	Drop-down<br>1 Internally developed<br>2 Philips<br>3 Epic<br>4 IMDLive<br>5 Bernoulli<br>6 Other
45	oth_ven	Please specify and describe "other" vendor.	Notes Box, Branching logic expression: [interaction_v2_v2]=4
46	choosesystem	What were the two most important factors in choosing a Tele-Critical Care system?	Checkboxes<br>1 Vendor's reputation and level of experience

			2 Level of technical support<br>3 Flexible cost structure regarding initial capital costs<br>4 Technological scalability and interoperability with various electronic health records, devices and servers<br>5 Transparent and open collaboration opportunities among various Tele-Critical Care providers and centers regardless of vendor<br>6 Other
47	otherfactor	Specify and describe "other" factors?	Notes Box, Branching logic expression: [choosesystem(6)] = '1'
48	lenght_inter_v2_v2	How many Tele-Critical Care interactions do you estimate will occur per shift?	Drop-down<br>1 0-20<br>2 21-50<br>3 51-80<br>4 81-110<br>5 111-150<br>6 More than 150
49	effect_v2	What are the three most important ways you will evaluate the effectiveness of Tele-Critical Care services?	Checkboxes<br>1 We do not assess effectiveness<br>2 Length of stay/days saved<br>3 Best-practice outcomes<br>4 Mortality/lives saved<br>5 ICU Readmission<br>8 Efficient bed utilization<br>9 Staff retention<br>10 Patient experiences<br>11 Shared decision-making<br>12 Cost savings
50	termination	Section Header: *Had Tele-Critical*	Checkboxes

		Care in the past (Branching logic expression: [utlz]=4)<br>In your opinion, what were the two most important reasons for the termination of the Tele-Critical Care program?	1 Costs<br>2 No appreciable need for Tele-Critical Care services<br>3 Change in workflow rendering Tele-Critical Care obsolete<br>4 Change in ownership of the unit<br>5 System change in a hospital system<br>6 Other
51	termination_text	Please specify and describe "other" reason for termination of Tele-Critical Care services.	Notes Box, Branching logic expression: [termination(6)] = '1'
52	terminationoper	How difficult was it to terminate services from an operational standpoint?	Slider, Slider labels: Very difficult, As expected, Much easier than expected
53	satisfaction	Section Header: *Currently using Tele-Critical Care services (Branching logic expression: [utlz]=6)*<br>How satisfied are you with Tele-Critical Care services?	Slider, Slider labels: Very dissatisfied, Neutral, Very Satisfied
54	long	For how many years have you been utilizing Tele-Critical Care services?	Number Box (Decimal), Min: 0, Max: 50
55		How often do you use each of these Tele-Critical Care models	Matrix Of Fields
56	item1_sec7	Continuous (24/7)	Matrix Of Fields: How often do... Radio Buttons<br>1 Most often used<br>2 Often used<br>3 Seldom used<br>4 Never used
57	item2_sec7	Reactive (in response to an acute physiologic issue)	Matrix Of Fields: How often do... Radio Buttons<br>1 Most often used<br>2 Often used

			3 Seldom used<br>4 Never used
58	item3_sec7	On-demand critical care consultation	Matrix Of Fields: How often do... Radio Buttons<br>1 Most often used<br>2 Often used<br>3 Seldom used<br>4 Never used
59	item4_sec7	Scheduled Tele-Critical Care rounds at a particular time	Matrix Of Fields: How often do... Radio Buttons<br>1 Most often used<br>2 Often used<br>3 Seldom used<br>4 Never used
60	item5_sec7	Utilization of Clinical Decision Support – Attention and treatment response prn to algorithmic severity/acuity scores, smart alerts, deterioration scores	Matrix Of Fields: How often do... Radio Buttons<br>1 Most often used<br>2 Often used<br>3 Seldom used<br>4 Never used
61	relationship	How would you describe the relationship between Tele-Critical Care and bedside ICU Clinicians?	Slider, Slider labels: ICU driven, ICU and Tele-Critical Care driven equally, Tele-Critical Care driven
62	interaction	Do you physically, rather than remotely, interact with Tele-Critical Care Clinician staff?	Drop-down<br>1 Meet routinely in-person<br>2 Meet in-person on an ad hoc basis<br>3 Tele-Critical Care providers work in both locations<br>4 No in-person interaction at all
63	lenght_inter	How many times does interaction with Tele-Critical Care services occur per shift?	Drop-down<br>1 0-20<br>2 21-50

			3 51-80<br>4 81-110<br>5 111-150<br>6 More than 150
64	ration	What is the optimal established patient-to-staff ratio for the number of Tele-Critical Care beds covered per provider?	Drop-down<br>1 0-50<br>2 51 - 100<br>3 101 - 150<br>4 151 - 200<br>5 201 - 250<br>6 251 - 300<br>7 More than 300
65	effect_2	How do you evaluate the effectiveness of Tele-Critical Care services? Select all that apply.	Checkboxes<br>1 We do not assess effectiveness<br>2 Length of stay/days saved<br>3 Best-practice outcomes<br>5 Mortality/lives saved<br>6 ICU readmission<br>8 Efficient bed utilization<br>9 Staff retention<br>10 Patient experience<br>11 Shared decision-making
66	satisfaction_v2	Section Header: *Currently providing Tele-Critical Care services (Branching logic expression: [utlz]=5)*<br>How satisfied are you with providing Tele-Critical Care services?	Slider, Slider labels: Very dissatisfied, Neutral, Very satisfied
67	long_v2	For how many years have you been providing Tele-Critical Care services?	Number Box (Decimal), Min: 0, Max: 50
68		Please indicate your Tele-Critical Care delivery model preferences	Matrix Of Fields

69	item1_sec8	Continuous (24/7)	Matrix Of Fields: Please indic...<br>Radio Buttons<br>1 Most preferred<br>2 Somewhat preferred<br>3 Neutral<br>4 Not preferred
70	item2_sec8	Reactive in response to an acute physiologic issue	Matrix Of Fields: Please indic...<br>Radio Buttons<br>1 Most preferred<br>2 Somewhat preferred<br>3 Neutral<br>4 Not preferred
71	item3_sec8	On-demand critical care consultation	Matrix Of Fields: Please indic...<br>Radio Buttons<br>1 Most preferred<br>2 Somewhat preferred<br>3 Neutral<br>4 Not preferred
72	item4_sec8	Scheduled Tele-Critical Care rounds at a particular time	Matrix Of Fields: Please indic...<br>Radio Buttons<br>1 Most preferred<br>2 Somewhat preferred<br>3 Neutral<br>4 Not preferred
73	item5_sec8	Utilization of Clinical Decision Support – Attention and treatment response prn to algorithmic severity/acuity scores, smart alerts, deterioration scores	Matrix Of Fields: Please indic...<br>Radio Buttons<br>1 Most preferred<br>2 Somewhat preferred<br>3 Neutral<br>4 Not preferred
74	relationship_v2	How would you describe the relationship between Tele-Critical Care and bedside ICU partners?	Slider, Slider labels: Bedside ICU driven, Bedside ICU and Tele-Critical Care driven equally, Tele-Critical Care driven

75	length_inter_v2	How many times does interaction with Tele-Critical Care services occur per shift?	Drop-down<br>1 0-20<br>2 21-50<br>3 51-80<br>4 81-110<br>5 111-150<br>6 More than 150
76	ration_v2	In your opinion, what is the optimal patient-to-clinician staff ratio for the number of Tele-Critical Care beds covered per provider?	Drop-down<br>1 0-50<br>2 51 - 100<br>3 101 - 150<br>4 151 - 200<br>5 201 - 250<br>6 251 - 300<br>7 More than 300
77	workteam	Who simultaneously works with you in Tele-Critical Care during the shift? Select all that apply.	Checkboxes<br>1 Another physician<br>2 Multiple physicians<br>3 Non-physician provider(s)<br>4 Nurse(s)<br>5 Pharmacist(s)<br>6 Data coordinator(s)<br>7 Fellow(s)/Trainee(s)<br>8 Administrator(s)
78	effect	How do you evaluate the effectiveness of Tele-Critical Care services? Select all that apply.	Checkboxes<br>1 We do not assess effectiveness<br>2 Length of stay/days saved<br>3 Best-practice outcomes<br>5 Mortality/lives saved<br>6 ICU readmission<br>8 Efficient bed utilization<br>9 Staff retention

			10 Patient experience<br>11 Shared decision-making
